# Modeling primary immunotherapy resistance in metastatic bladder cancer: a syngeneic, bioluminescent mouse model

**DOI:** 10.1186/s12935-025-04117-x

**Published:** 2026-01-08

**Authors:** Dongbo Xu, Justine J. Jacob, Kyle Wieczorek, Li Wang, Han Yu, Jianmin Wang, Bo Xu, Ahmed A. Hussein, Khurshid Guru, David W. Goodrich, Qiang Li

**Affiliations:** 1 Department of Urology, Roswell Park Comprehensive Cancer Center, Buffalo, NY USA; 2 Department of Biostatistics and Bioinformatics, Roswell Park Comprehensive Cancer Center, Buffalo, NY USA; 3 Department of Pathology, Roswell Park Comprehensive Cancer Center, Buffalo, NY USA; 4 Department of Pharmacology and Therapeutics, Roswell Park Comprehensive Cancer Center, Buffalo, NY USA

**Keywords:** Bladder cancer, Mouse model, Metastasis, Immunotherapy, Anti–PD-1

## Abstract

Approximately 90% of bladder cancer deaths are due to distant metastases rather than local tumor effects. The current first-line systemic treatment for metastatic bladder cancer (mBC) is chemoimmunotherapy or immunotherapy with pembrolizumab plus enfortumab vedotin (EV). However, most mBC patients treated with chemoimmunotherapy or pembrolizumab/EV do not respond or eventually relapse, highlighting the critical need for robust immunocompetent animal models to elucidate the mechanisms of primary and acquired resistance. We previously generated a syngeneic murine cell line CMV-TRP (triple knockout of *Trp53*, *Rb1*,* Pten*) via ex vivo transduction with adenovirus (Ad5CMVCre). To establish an mBC model, the TKO cells were effectively labeled with a lentiviral luciferase and GFP double-expressing reporter and injected into tail veins of C57 BL/6J mice. Tail vein injection of TKO cells reliably established distant metastases with lung and bone lesions. In immunotherapy experiments, mice injected with TKO-labeled cell lines were randomly treated with an anti–PD-1 or control IgG2a antibody. All mice developed lung and/or bone (hind limb or sacrum) metastases. There was no difference in tumor bioluminescence between the control group and anti–PD-1 group (median proton/second 6.94 × 10^8^ vs. 4.32 × 10^8^, *p* = 0.85). Kaplan-Meier analysis showed no difference in median survival between the control group and anti–PD-1 group (19 days vs. 20 days, *p* = 0.47). Histology and immunohistochemical profile of lung and bone metastases revealed high-grade basal-like urothelial carcinoma, closely resembling the profile observed in subcutaneous tumor. No significant changes in immune cell infiltrations (CD4^+^, CD8^+^, or F4/80^+^) between groups may explain anti–PD-1 immunotherapy resistance. Therefore, the novel TKO metastatic model represents a useful and reproducible tool for studying tumor-cell dissemination, bone/lung metastasis, and the underlying mechanisms of anti–PD-1 immunotherapy resistance.

**Supplementary Information:**

The online version contains supplementary material available at 10.1186/s12935-025-04117-x.

## Introduction

Bladder cancer is the fourth most common malignancy in men and causes an estimated 18,070 deaths annually in the United States [1]. In 2022, bladder cancer was the 9th most common and 13th most deadly cancer worldwide [2]. Metastatic bladder cancer (mBC) accounts for the majority of bladder cancer deaths [3]. Approximately 5% of bladder cancer patients present with metastatic disease at diagnosis, and 30–50% of patients with localized bladder cancer eventually develop metastases during the tumor progression [4]. mBC has a very poor prognosis, with the 5-year survival rate of 5%–10% [5, 6]. In the past four decades, chemotherapy (GC: gemcitabine–cisplatin or MVAC: methotrexate, vinblastine, adriamycin, and cisplatin) has been the standard first-line treatment for mBC. However, mBC patients treated with chemotherapy had a median survival of 12–15 months, only 3–6 months if not undergoing treatment [6, 7]. A report on 7543 patients with mBC revealed that the most common sites included lymph nodes (25%), bone (24%), urinary (23%), lung (19%), liver (18%), and brain (3%). In another cohort of 1862 mBC patients, bone metastases were most frequent (43%), followed by lung metastases (39%) [8]. Multiple metastatic sites were observed in 29% of mBC patients [9].

Recent advances in anti–PD1/PD-L1 immunotherapy have transformed the therapeutic landscape in mBC. The current first-line systemic treatments for mBC include chemoimmunotherapy or the combination of pembrolizumab and enfortumab vedotin (EV) [10, 11]. However, some mBC patients do not respond to chemoimmunotherapy (23.5% progression-free patients treated with Nivolumab plus Gemcitabine–Cisplatin in 24 months), and most patients eventually relapse, highlighting the critical need for robust immunocompetent animal models to elucidate the mechanisms of primary and acquired resistance [11]. The molecular mechanism of metastases and treatment resistance in mBC has been understudied, likely due to a lack of clinically relevant preclinical models [12, 13]. Compared to in vitro cell cultures, mouse models recapitulate the complex tumor microenvironment and overcome the limitation of the artificial nature of the culture system [14]. However, the establishment, assessment, and imaging of animal models of mBC is challenging. Currently, intravesical delivery of cancer cells could lead to invasive orthotopic growth and metastases [15, 16]. A first optical-imaging-based preclinical model using intravesical delivery of human UMUC-3luc2 cells was established for assessing the tumor growth and lymph/lung metastases [3]. Flank subcutaneous injection of PDX (patient-derived xenograft) BL0293F also results in liver metastases imaged by an effective non-contrast T2w MRI technique [17]. A more efficient approach for modeling mBC involves delivering cells directly into blood circulation by lateral tail vein [18] or left cardiac ventricle [19]. Mouse tail vein and caudal artery injections for modeling bone metastases have been shown to require relatively low technical proficiency, offer high delivery efficiency, and induce less acute death of mice, compared to intracardiac injection as a gold standard in the past [20]. Together, there is a clear need for a reliable and efficient mBC model in immunocompetent mice that enables real-time tumor tracking and drug responses evaluation to immunotherapy.

Immunocompetent mouse models are especially valuable for studying the mechanisms that drive responses to immunotherapy. In the TCGA cohort of 412 patients, mutations in TP53, RB1/CDKN2A, and PTEN/PIK3CA occurred in approximately 50%, 65%, and 35% of cases, respectively. Based on this mutational pattern, we generated triple-knockout (TKO) cells to model the most common alternative pathway and capture a clinically relevant pattern of bladder tumorigenesis [21]. Our lab previously developed a stable TKO organoid model with the mutations of tumor suppressors *Trp53*, *Pten*, and *Rb1* using ex vivo transduction and grafted it on immunocompetent C57 BL/6J (B6) mice [22]. Molecular and histologic analyses of allografts confirmed high-grade urothelial carcinoma with squamous differentiation and a basal-like subtype. Immunotherapy (anti–PD-1 antibody) on this subcutaneous tumor exhibited differential responses. Responders (33%) exhibited a higher levels of immune cell infiltration, including increased infiltration of macrophages and T cells, compared to non-responders [23]. Here, we generated a 2D cell line from previously established TKO organoids and labeled the cells with GFP and luciferase for in vivo and in vitro tracking. These labeled TKO cells were injected into the tail vein to establish mBC models. Bioluminescence was successfully detected by In Vivo Imaging System (IVIS) at sites of lung and/or bone metastases. After establishing this reliable metastatic model, we tested immunotherapy response using an anti–PD-1 antibody. The model exhibited primary resistance to anti–PD-1 treatment, thereby enabling the establishment of a novel immunotherapy-resistant mouse model of mBC. This stable model closely recapitulates the clinical course (lung and bone metastasis) of advanced bladder cancer and provides a valuable platform for investigating the biological process and mechanisms underlying immunotherapy resistance.

## Materials and methods

### 2D cell line culture

The TKO (triple gene knockout of *Trp53*,* Rb1*,* Pten*) cell line CMV-TRP was isolated from previously established TKO organoid culture [23], and passaged as a 2D culture in complete culture medium. The complete culture medium consisted of Mammary Epithelial Cell Growth Medium supplemented with the manufacturer’s growth factor kit (Lonza CC-3150), 10% charcoal-stripped fetal bovine serum (Thermo Fisher), 1% GlutaMAX (Gibco), 100 µg/mL Primocin (InvivoGen), and 10 µM freshly prepared Y-27632 (Selleckchem). HEK293T cells were cultured in DMEM (Corning) with 10% FBS and 1x penicillin-streptomycin. Cells were cultured at 37 °C in a humidified incubator with 5% CO_2_.

### Lentivirus production and transduction

Plasmid pFUGW-Pol2-ffLuc2-eGFP was a gift from Glenn Merlino (Addgene plasmid # 71394) [24]. Lentivirus plasmid pFUGW-Pol2-ffLuc2-eGFP (25 µg) was co-transfected with packaging plasmid psPAX2 (6.25 µg) and envelope plasmid pMD2.G (6.25 µg) in HEK293T cells in a 100 mm culture plate using Lipofectamine 3000 reagent. Lentivirus particles were concentrated using PEG-it Virus Precipitation Solution (System Biosciences). Lentivirus particles were resuspended in phosphate-buffered saline (PBS) and frozen in −80 °C freezer.

For lentivirus transduction, organoid cells were digested and resuspended in 0.5 mL culture medium mixed with lentivirus and 2.5 µL TransDux™ reagent (System Biosciences) in a 24-well plate. The plate was centrifuged at 300 G for 30 min at room temperature by spinoculation for enhancing transduction efficacy, then moved into an incubator at 37 °C for 1 h. Organoid cells were collected and re-plated in 70% Matrigel in a 6-well plate for culturing. For the 2D cell line, CMV-TRP cells were transduced with lentivirus using TransDux™ reagent for 48 h incubation following the manufacturer’s protocol, and the transfected cells were confirmed by a GFP reporter.

### FACS-based GFP sorting

The plasmid pFUGW-Pol2-ffLuc2-eGFP construct lacks an antibiotic resistance gene; therefore, transfected cells cannot be selected using antibiotic-based selection. To enrich the GFP-expressing cells, 2 million transfected CMV-TRP cells were collected and filtered through a 40-µm cell strainer (Corning) to ensure single-cell resolution. GFP-positive cells were sorted using a SONY MA900 cell sorter equipped with 488-nm and 561-nm lasers. Forward scatter (FSC) and Back scatter (BSC) profiles were used to exclude debris and doublets. Sorted cells were used immediately for culturing in the incubator.

### Luciferase reporter assay

2D culturing cells were collected and lysed with 5X reporter lysis buffer (Promega) following the manufacturer’s protocol. Lysate (20 µL) was added into a well of a 96-well plate, then mixed with 100 µL/well luciferase substrate solutions in the well [10 µL 10 mM D-luciferin, 15 µL 200 mM ATP, 1.35 mL buffer (100 mM Tris acetate, pH 7.8, 10 mM Magnesium acetate, 1 mM EDTA)]. Lastly, the plate was read by Veritas microplate luminometer (Turner Biosystems).

### Mouse model by lateral tail vein injection

All animal procedures were approved by the Institutional Animal Care and Use Committee (IACUC) at Roswell Park Comprehensive Cancer Center (RPCCC). C57 BL/6J (B6) male mice of 10 weeks were obtained from Jackson Laboratory (stock #000664). Stably transfected cells were passaged in immunocompetent mice by flank subcutaneous injection. Two million cells were resuspended in 50% Matrigel/PBS and subcutaneously injected into the right flank of B6 mice. All mice were euthanized by CO_2_ inhalation followed by cervical dislocation. Tumor was harvested and dissociated into single-cell suspensions once it reached approximately 1 cm in diameter. Tumor cell suspensions were obtained and processed following the reported tumor dissociation protocol [22]. Cells were passed through a 40-µm cell strainer prior to tail vein injection to ensure a single-cell suspension.

Freshly dissociated 8 × 10^5^ tumor cells in 100 µL saline were injected into mouse lateral tail veins using an insulin syringe with 30G ½ needle and a mouse restrainer device. After 12 days of post-injection, the injected mice were monitored by IVIS (Perkin Elmer) imaging twice per week. Upon tumor detection (whole-body bioluminescence reaches around 1 × 10^6^ photons/sec.), mice with comparable signal intensity were assigned randomly to receive either anti–PD-1 (BioXcell, RMP1-14, 200 µg, intraperitoneally twice weekly, Catalog: BP0146) (*n* = 8) or control IgG2a (BioXcell, 2A3, 200 µg, intraperitoneally twice weekly, Catalog: BP0089) (*n* = 6) treatment. The treatment period lasted for three weeks. These antibodies are the same batch product purchased from company, which was used in previous TKO subcutaneous model study [23]. The mice were monitored for overall survival, serial imaging, and analyses of tumor metastasis at the study endpoint.

### Bioluminescent imaging

For bioluminescent imaging, lesions in mice were detected by whole body in vivo by IVIS (Perkin Elmer). Anesthetized mice received an intraperitoneal injection of sterile 150 mg/kg D-Luciferin solution (15 mg/mL) in PBS and imaged for maximum luciferase signal at 15 min post-injection. Luminescence was detected by IVIS for all experimental mice in same period, and the following conditions were used for image acquisition: open emission filter, binning = medium: 8, field of view 23.2 cm, and f/stop = 1. Display and image analysis were performed using Living Image Software (Perkin Elmer).

### Histological analysis

Mice were euthanized at the moribund stage for tumor metastatic tissue collection. The lung and hind limbs or other bones with metastatic sites were excised, fixed in 4% paraformaldehyde prepared in PBS, and embedded in paraffin. Tissue sections of 5 μm thickness were prepared for hematoxylin and eosin (H&E) staining and immunohistochemistry. Metastatic tumors were stained for H&E and immunohistochemistry (IHC) staining with urothelial markers (CK7, p63, GATA-3, CK5, CK8, and Uroplakin 3) and immune biomarkers (CD4, CD8, PD-1, F4/80). Immunofluorescent (IF) staining and IHC were performed as previously described [25].

### Single-cell RNA data analysis

Single cell RNA sequencing of subcutaneous TKO tumors treated with and without PD-1 have been previously described and deposited in GEO as GSE200139 [23]. All cells were initially clustered using the Louvain algorithm with a shared nearest neighbor (SNN) graph and t-distributed Stochastic Neighbor Embedding (t-SNE) was used for visualization of the merged datasets using Seurat. Broad cell type annotations were then implemented based on established gene markers from the literature and reference mapping predictions using SingleR Immgen Mouse Atlas. Scaled normalized expression of clinically relevant immune checkpoint target genes from Shi et al. [26] were assessed between control and treatment group, and unscaled normalized expression of the gene list was assessed in control IgG2a tumor.

### Statistical analysis

All statistical analyses were conducted using GraphPad Prism version 10. All data are presented as median with the range. The survival curve was performed using Kaplan-Meier analysis and presented as median with the range. All comparisons between two groups were performed using the non-parametric Mann–Whitney test. Statistical significance was defined as ns (not significant), *p* < 0.05 (*), *p* < 0.01 (**), and *p* < 0.001 (***).

## Results

### TKO cells labeled with a luciferase and GFP double-expressing reporter

**Fig. 1:**
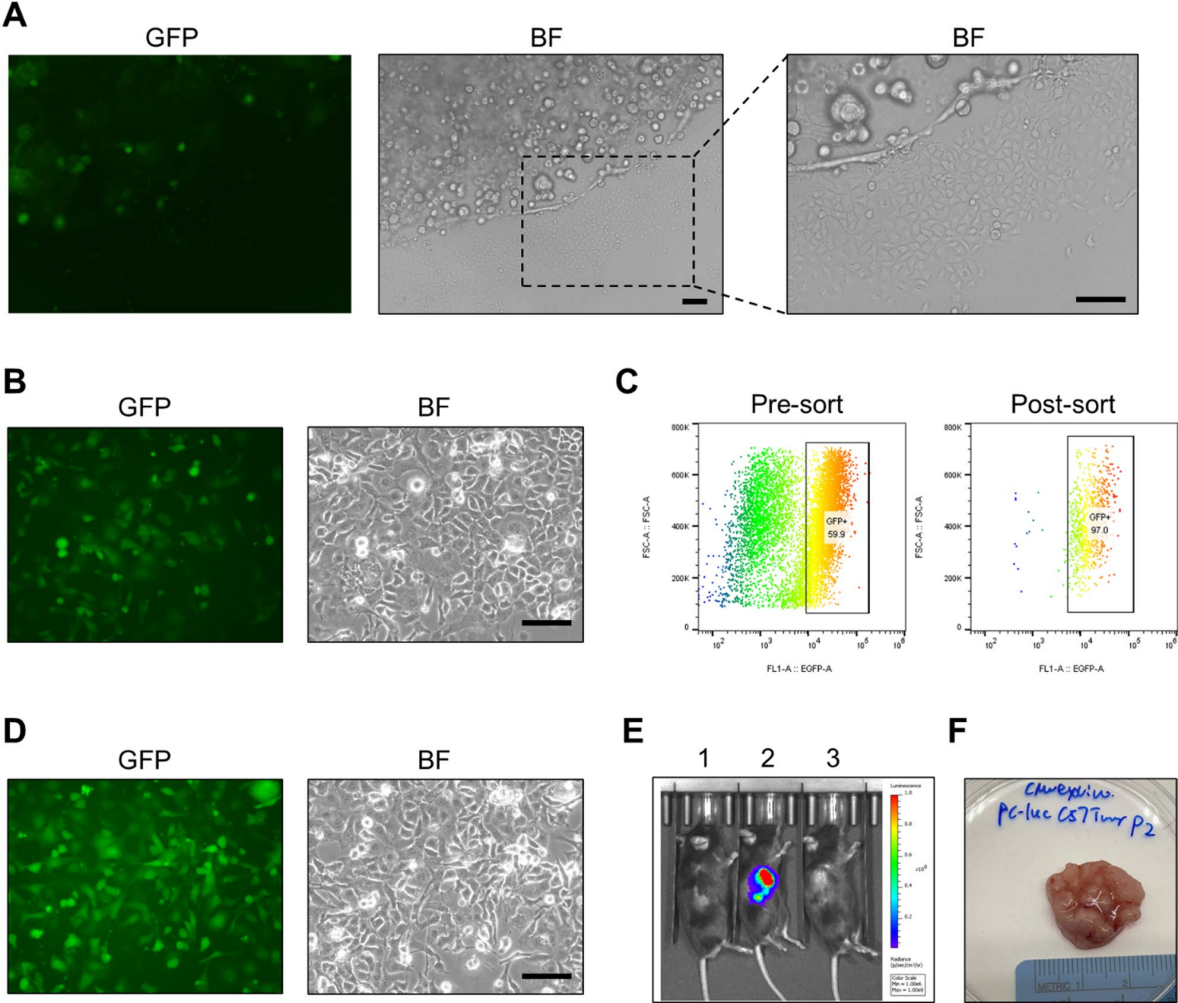
TKO cells were labeled with a luciferase and GFP double-expressing reporter. (**A**) GFP fluorescence and brightfield (BF) microscopy images of TKO cells in 3D organoid culture following pFUGW-Pol2-ffLuc2-eGFP lentiviral transduction. (**B**) Representative GFP fluorescence and BF images of transduced TKO cells in 2D culture by second lentivirus transduction. (**C**) Flow cytometry plots showing GFP percentage in pre-sorting and post-sorting 2D CMV-TRP-luc cells by FACS. (**D**) The GFP fluorescence and BF images of culturing 2D CMV-TRP-luc cells after GFP sorting. (**E**) IVIS bioluminescence images in B6 mice bearing subcutaneous tumors. Maximum luciferase signals were recorded at 15 min post-injection of D-Luciferin solution. Mice 1 is normal mice, mice 2 is bearing a tumor from 2D CMV-TRP-luc cells, and mice 3 bears a tumor from the first-time transduced 3D organoid CMV-TRP. (**F**) The gross picture of a subcutaneous tumor from 2D CMV-TRP-luc cells in a B6 mouse. Scale bar: 100 μm

TKO organoids established previously [23] were generated from the bladder urothelium of triple-floxed mice (*Trp53*^*fl/fl*^ *Pten*^*fl/fl*^ *Rb1*^*fl/fl*^) by transducing ex vivo with adenovirus Ad5CMVCre. TKO organoids were confirmed with mutations on genes of *Trp53*,* Rb1*,* Pten* and exhibited basal-like urothelial carcinoma. By spinoculation with lentivirus (pFUGW-Pol2-ffLuc2-eGFP), 3D organoid cultures successfully exhibited detectable GFP expression under an immunofluorescence microscope (Fig. 1A). However, only a small proportion of GFP-positive cells (~ 10%) were observed, indicating low transduction efficiency. In 3D organoid culturing, some cells adhered to plate at the edges of the Matrigel domes and began to grow as a 2D monolayer. Given that 2D cultures are easier to maintain and transduce, the Matrigel was removed to propagate adherent 2D TKO cells. A second lentiviral transduction was then performed on the 2D TKO cells, resulting in a marked increase in GFP-positive cells (60%) (Fig. 1B). To further enrich the transduced population, a single-cell suspension was sorted by Fluorescence-Activated Cell Sorting (FACS) based on GFP expression. As shown in Fig. 1C-D, the proportion of GFP-positive cells increased from 60% before sorting to over 97% after sorting. The bioluminescence signal from the sorted cell lysate was confirmed and detected by luciferase reporter assay. The sorted early-passage 2D TKO cells (designated CMV-TRP-luc) were then subcutaneously injected into the right flank of immunocompetent B6 mice to confirm in vivo imaging. By day 21 post-injection, a strong bioluminescent signal (2 × 10^9^ total flux photons/second) was detected in mice bearing tumors derived from the 2D transduced cells (Fig. 1E), whereas tumors from the organoid-transduced cells showed no detectable signal. Tumor with strong bioluminescence was harvested (Fig. 1F), dissociated into a single-cell suspension, cryopreserved, or used for in vivo passaging on B6 mice. Therefore, these TKO CMV-TRP cells were successfully labeled with a luciferase and GFP double-expressing reporter, and reliably tracked both in vitro and in vivo, offering a powerful tool for establishing and noninvasively tracking metastatic bladder cancer progression in mouse models.

### *In vivo* imaging analyses of mBC tumors in C57 BL/6J mice

**Fig. 2:**
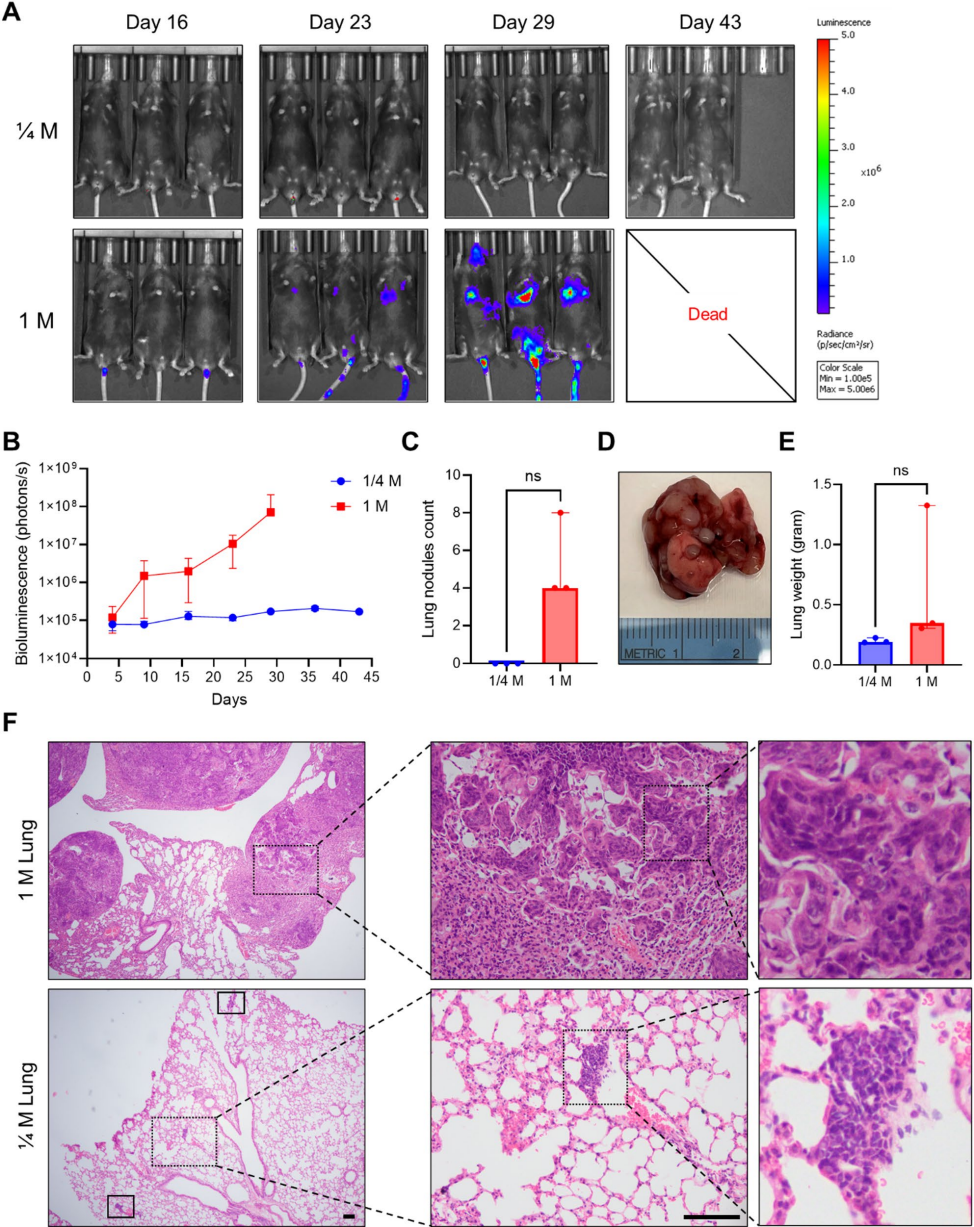
In vivo imaging analyses of metastatic tumors with different cell numbers via tail vein injection. (**A**) Representative IVIS images and intensity of whole-body region of interest in mice injected with 0.25 (top row) or 1 million (bottom row) cells at day 16, 23, 29, 36, and day 43. Maximum luciferase signals were recorded at 15 min post-injection of D-Luciferin solution. (**B**) Quantitative analysis and bioluminescence curve of whole body in mice after tail vein injections with 0.25 or 1 million cells. Signal intensity was shown as days after cell injection. Curves were shown as median with range. (**C**) Number of developed lung nodules in mice with 0.25 or 1 million cells tail vein injection. Plot data were presented as median with range, *p* = 0.1. (**D**) The gross picture of lung nodules from a representative mouse with 1 million cells injection. (**E**) The weight of the whole lung tissue in mice. Plot data were presented as median with range, *p* = 0.1. (**F**) The histology examination of lung nodules metastasis in a mouse with 1 million cells injection or 0.25 million cells. Scale bar: 100 μm. ns: not significant

TKO tumors derived from CMV-TRP-luc cells exhibiting strong bioluminescence were serially passaged in B6 mice. To determine the metastatic potential of CMV-TRP-luc cells, freshly dissociated CMV-TRP-luc cell suspensions from subcutaneous tumors were injected via the lateral tail vein at doses of 0.25 million or 1 million cells per mouse. No acute mortality was observed in any mice. Notably, all three mice injected with 1 million cells showed transient inactivity immediately following the injection but recovered within 15 min, likely due to the high cell load in the tail vein. IVIS imaging was conducted weekly post-injection. In the 1 million cells group, all mice developed strong bioluminescence signals (1.0 × 10^7^ photons/second, median) in whole-body region by day 23 (Fig. 2A-B), which increased to 0.7 × 10^8^ p/s (median with range 0.6–2.1 × 10^8^ p/s) by day 29. In contrast, mice injected with 0.25 million cells showed only background-level signals (~ 1 × 10^5^ p/s) through day 43. All three mice from the 1 million cells group and one mouse from the 0.25 million cells group were euthanized on day 29 for tumor collection, while the remaining two mice from the 0.25 million cells group were euthanized on day 43. Four tumor nodules (median with range 4–8) were identified in the lungs of mice receiving 1 million cells only (Fig. 2C and D), and lung weights [median 0.35 g with the range 0.31–1.33 g] were increased in these mice compared to normal lung tissue from mice with 0.25 million cells [median 0.19 g with the range 0.19–0.23 g], although statistical significance was not reached (*p* = 0.1), likely due to the limited sample size (Fig. 2E). Interestingly, one mouse also developed bone metastasis at the sacrum. Histological analysis of lung lesions confirmed multifocal metastases with histology consistent with previously characterized TKO subcutaneous urothelial tumors (Fig. 2F) [22, 23]. Notably, only one mouse from the 0.25 million cells group exhibited small metastatic lesions detectable only microscopically, although no visible metastatic nodules were observed in any of the three mice in this group (Fig. 2F). Taken together, tail vein injection of 1 million CMV-TRP-luc cells reliably induces lung metastasis within one month, offering a reproducible and clinically relevant model for studying bladder cancer metastasis and therapeutic responses to immunotherapy.

### mBC TKO tumors exhibit primary resistance to anti–PD-1 treatment

TKO subcutaneous tumors exhibited a heterogeneous response to anti–PD-1 antibody treatment, with the majority of tumor-bearing mice (6 out of 9) showing no response at all [23]. Using this mBC model in immunocompetent mice, we investigated the therapeutic response of the metastatic tumors from TKO CMV-TRP-luc cells. To achieve a high tumor take rate in a larger cohort while minimizing complications related to tail injection, we determined that 0.8 million cells represented the optimal dose. This dosage avoided the low take rate seen with 0.5 million cells and the injection-related complications observed with 1.0 million cells. Two mice died immediately following the injection. IVIS imaging was performed twice weekly thereafter. The 14 surviving mice were randomly selected for treatment with either control IgG2a (*n* = 6) or anti–PD-1 antibody (*n* = 8), administered twice weekly beginning when whole-body bioluminescence reached ~ 1 × 10^6^ photons/sec.

**Fig. 3:**
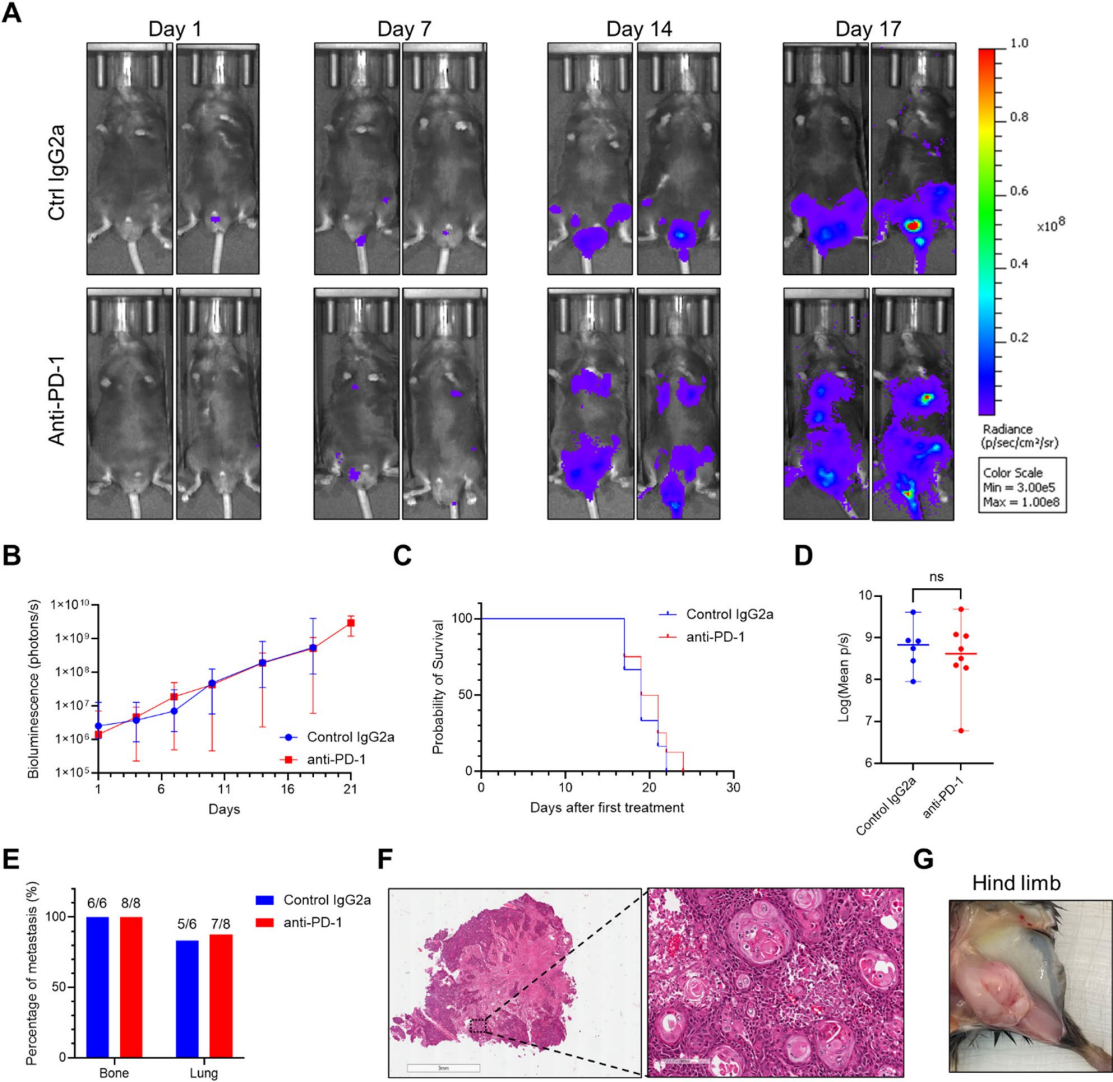
TKO mBC tumors exhibit primary resistance to anti–PD-1 immunotherapy. (**A**) Representative IVIS images and intensity of whole-body region of interest in mice treated with Control IgG2a (top row) and anti–PD-1 (bottom row) antibodies. (**B**) Quantitative analysis of bioluminescence of whole body in mice after the first treatment. The first treatment was started once the bioluminescence intensity of the whole body reached 1 × 10^6^ p/s. Maximum luciferase signals were recorded at 15 min post-injection of D-Luciferin solution. Signal intensity was shown as days after receiving treatment. Curves were shown as median with range. (**C**) Kaplan-Meier analysis on mice received anti–PD-1 (*n* = 8) and control IgG2a (*n* = 6) treatment (*p* = 0.47). Control IgG2a group median survival is 19 days (range 17–22 days) and anti–PD-1 group median survival is 20 days (range 17–24 days). (**D**) Bioluminescence intensity of terminal tumors between groups (*p* = 0.85). Plot data were presented as median with range. (**E**) Metastatic lesion locations of bone or lung between groups. (**F**) The histology examination of sacrum bone metastasis in a mouse. (**G**) The gross picture of hind limb bone metastasis in a mouse. ns: not significant

Mice were euthanized or dead between days 17 and 24 after the first treatment due to respiratory or motor impairments caused by metastatic tumor burden. All 14 mice developed detectable metastases, confirming a 100% metastasis take rate via tail vein injection. Anti–PD-1 treatment failed to prevent metastatic progression, as reflected in both signal intensity and metastatic distribution (Fig. 3A-B). At day 18, the median whole-body bioluminescence of anti–PD-1 treatment group reached 5.22 × 10^8^ photons/sec, whereas the control group reached 5.54 × 10^8^ photons/sec. Notably, all mice in both groups became moribund around day 18 after the first treatment, whereas two mice in the anti–PD-1 group reached moribund status on day 21. Kaplan-Meier analysis showed no significant difference in median survival between the control and anti–PD-1 groups [19 days (range 17–22 days) vs. 20 days (range 17–24 days), *p* = 0.47; Fig. 3C]. Terminal bioluminescence also showed no significant difference between control group (median p/s 6.94 × 10^8^ with range 9.02 × 10^7^ – 4.08 × 10^9^) and anti–PD-1 group (median p/s 4.32 × 10^8^ with range 6.04 × 10^6^ – 4.81 × 10^9^, *p* = 0.85; Fig. 3D). Regarding metastatic distribution, 5 out of 6 mice in the control group and 7 out of 8 in the anti–PD-1 group developed lung metastases. All experimental mice developed bone metastases, with lesions detected in the hind limbs (5/6 in control, 6/8 in anti–PD-1), sacrum (2/6 in control, 4/8 in anti–PD-1), or both (Fig. 3E). Histological examination of sacral bone metastases (Fig. 3F) confirmed urothelial carcinoma morphology consistent with the original subcutaneous TKO tumors [22, 23]. A gross image of a hind limb metastatic lesion is shown in Fig. 3G. These results suggest that tail vein injection of TKO tumor cells in immunocompetent mice reliably establishes mBC lesions in the lung and bone, which are resistant to anti–PD-1 antibody treatment.

### Histopathology and immune microenvironment in mBC tumors

**Fig. 4:**
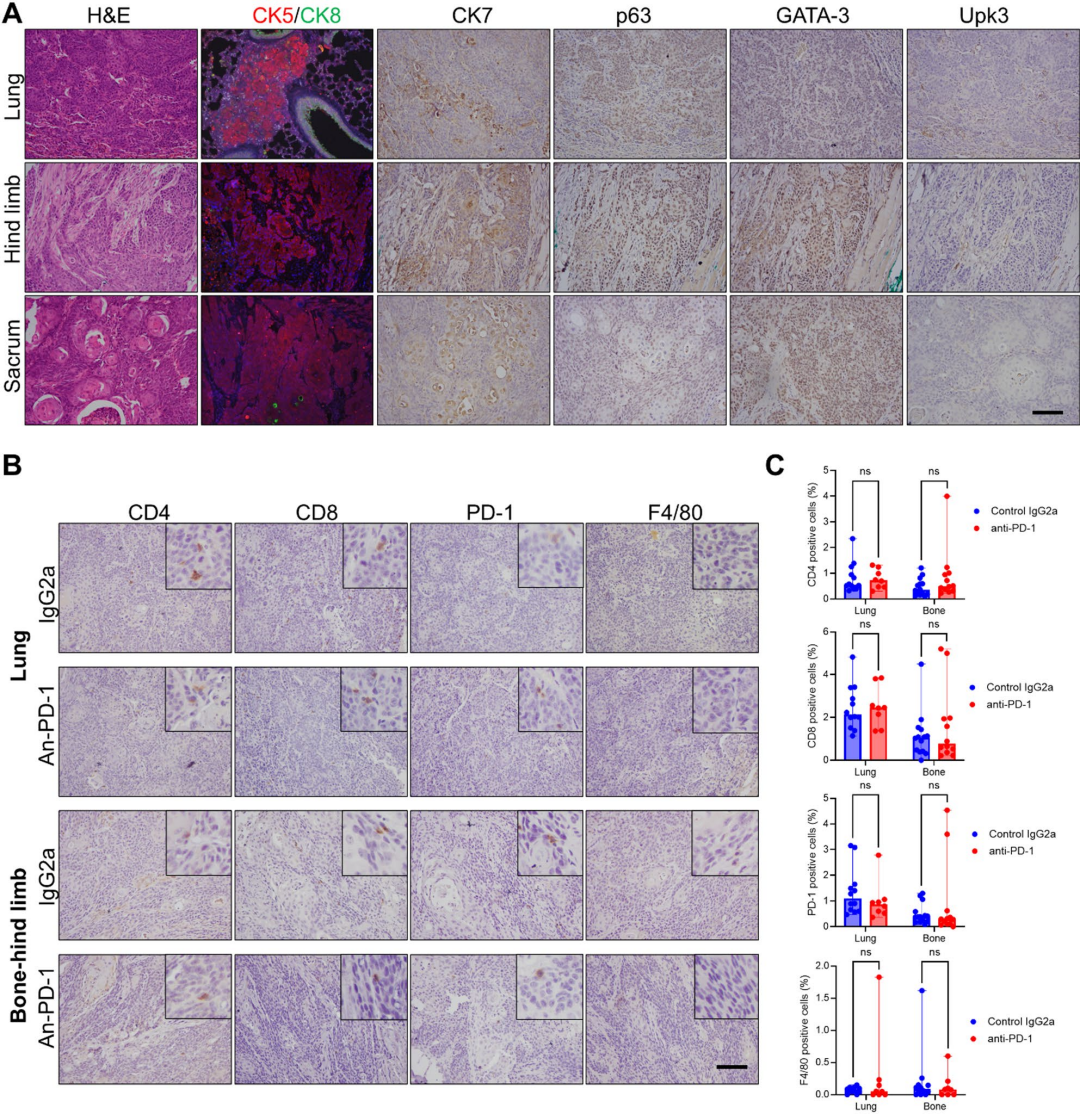
Immunostaining analysis and immune cell infiltration of lung and bone metastases. (**A**) H&E and immunohistochemical staining in lung, hind limb, sacrum metastatic sites with typical urothelial biomarkers (CK5, CK8, CK7, p63, GATA-3, and Upk3) in mice. (**B**) Immunohistochemical staining in metastatic tumor sites with representative immune cell biomarkers (CD4, CD8, PD-1, F4/80) from anti–PD-1 or Control IgG2a-treated mice. (**C**) Quantitative analysis of immune cell percentages within metastatic tumors (positive counts divided by all cells in the field). Each data point represents the calculated percentage from two random fields of representative tumors in each group. Bone tumors include hind limb and sacrum bone sites. Plot data were presented as median with range. Scale bar: 100 μm. ns: not significant

Most mice treated with either anti–PD-1 or control IgG2a antibody developed metastatic lesions across multiple anatomical sites, including the lungs, hind limbs, and sacrum. To assess the histopathological features of these lesions, we performed H&E staining along with IHC analyses for canonical urothelial markers (CK5, CK8, CK7, p63, GATA-3, and Upk3) across the various metastatic sites. All lesions demonstrated histological similarity to the original subcutaneous TKO tumors previously reported [22, 23], exhibiting positive staining for CK5, p63, and GATA-3, focal CK7 positivity, and negative staining for CK8 and Upk3 (Fig. 4A). These findings are consistent with a basal-like tumor phenotype across metastatic locations. Given the absence of significant differences in tumor burden or histological phenotype between the anti–PD-1 and control-treated groups, we next evaluated the immune landscape of these tumors to explore potential mechanisms of therapeutic resistance. We performed IHC staining for key immune markers, including CD4 (helper T cells), CD8 (cytotoxic T cells), PD-1 (immune checkpoint receptor), and F4/80 (macrophages). As shown in Fig. 4B-C and Supplementary Table S1, the control group exhibited minimal immune infiltration in tumors: CD4⁺ T cells constituted of the tumor-infiltrating population (median 0.55% in lung and 0.35% in bone), CD8⁺ T cells (median 2.14% in lung and 1.06% in bone), PD-1⁺ cells (median 1.09% in lung and 0.38% in bone), and F4/80⁺ macrophages (median 0.08% in lung and 0.09% in bone). Notably, the anti–PD-1-treated group showed no significant changes in immune cell infiltration [CD4^+^ (median 0.72% in lung and 0.50% in bone), CD8^+^ (median 2.45% in lung and 0.77% in bone), PD-1^+^ (median 0.87% in lung and 0.29% in bone), and F4/80^+^ (median 0.05% in lung and 0.08% in bone)] relative to controls, suggesting that the therapy failed to remodel the tumor immune microenvironment. These data indicate that the metastatic lesions in this model remain immunologically “cold” and intrinsically resistant to anti–PD-1 therapy, as evidenced by the lack of more immune cell recruitment or expansion.

To investigate the potential underlying mechanisms of immune resistance, we re-analyzed our previously reported single-cell RNA sequencing data [23] generated from the subcutaneous TKO (CMV-TRP) tumor model treated with anti–PD-1 therapy (supplementary Fig. S1A). There were more immune cells infiltrating TKO tumors that responded to anti–PD-1 treatment, whereas minimal changes in immune cell infiltration were observed in the TKO tumors that did not respond (Fig. S1B). To examine the landscape of immunotherapy target gene expression, we employed the 53-gene signature reported by Shi et al. in the TKO models, comprising 23 inhibitory and 30 stimulatory checkpoint genes [26]. Tumors that responded to anti–PD-1 treatment showed upregulation of immunotherapy target genes relative to control IgG and non-responders (Fig. S1C). However, unscaled normalized expression analysis in control TKO tumors showed low or absent immune checkpoint genes expression, including minimal PD-L1 (Cd274) on tumor cells, suggesting an intrinsic lack of immunotherapy targets (Fig. S1D). This paucity of immune checkpoint targets likely contributes to primary resistance to anti–PD-1 therapy. Future comprehensive profiling of untreated and treated metastatic lesions will be required to identify additional actionable resistance mechanisms and pathways that may allow conversion of these tumors to an immune-responsive state.

## Discussion

In this study, we established a robust and clinically relevant mBC model in immunocompetent mice using TKO urothelial carcinoma cells that are stably expressing a dual luciferase-GFP reporter. Tail vein injection of labeled TKO cells consistently induced reproducible lung and bone metastases with a high take rate, enabling non-invasive, longitudinal monitoring via IVIS imaging. This model reflects the three most commonly altered pathways (TP53, RB1, and PTEN/PIK3CA) in human bladder cancer and faithfully recapitulates the basal molecular subtype, characterized by multifocal dissemination. Anti–PD-1 treatment, failed to inhibit disease progression, improve survival, or alter immune cell infiltration. These findings highlight the translational relevance of our mBC model and support its utility as a platform for elucidating mechanisms of primary immunotherapy resistance and for evaluating novel combination treatment strategies.

Currently, numerous bladder cancer mouse models have been established, including spontaneous (carcinogen-based or GEMM) or transplantable (syngeneic or xenograft) models by orthotopically or heterotopically [27]. However, very few metastatic mBC models were generated. A patient-derived xenograft BL0293F was established in NSG mice as a reliable liver metastatic model (70% at 52 days) by implanting tumor fragments into right flank [17]. An orthotopic MB49 model on C57BL/6 mice exhibited an overall metastatic rate of 90% including lesions in lungs (23/26 mice), liver (22/26), ureter (11/26), and kidneys (5/26) [28]. Another lung metastatic model was established by performing serial tail-vein injections of human T24T urothelial carcinoma cells into nude mice, selecting and re-isolating derivative sublines (FL1, FL2, FL3) from pulmonary nodules to progressively enrich metastatic potential [29]. Nevertheless, previous models exhibit several limitations. Models of BL0293F and T24T are not syngeneic and not suitable for investigating the immune response and native microenvironment. Immunocompetent syngeneic mouse models, in which murine tumor cell lines are implanted into genetically matched hosts (e.g., C57BL/6), remain a cornerstone of preclinical immuno-oncology research. These models preserve intact tumor–immune interactions, enabling robust evaluation of checkpoint inhibitors, CAR-T therapies, and cancer vaccines [30].The MB49 model is a widely used syngeneic bladder cancer model; however, orthotopic implantation of MB49 cells yields tumors that lack key urothelial markers such as EpCAM and cytokeratins. As a result, this model fails to accurately recapitulate the molecular and phenotypic features of human urothelial carcinoma, limiting its translational relevance for studying disease biology and therapeutic response [31]. Furthermore, none of these studies incorporate real-time in vivo imaging to monitor metastatic tumor growth in mice. The exception is a study on MB49-luc conducted by Vandeveer et al. [32]. They used luciferase-labeled MB49 bladder cancer cells (MB49-luc) to monitor orthotopic tumor growth in vivo and to evaluate the antitumor efficacy of anti–PD-L1 antibody treatment. However, lung metastases were neither examined nor detected in this model. Interestingly, a recent study in 2024 by Desponds et al. reported a syngeneic *Trp53*^*fl/fl*^ *Pten*^*fl/fl*^ Luc-SIY⁺ inducible bladder cancer model, which exhibited a 12% metastatic rate by 12 weeks and resistance to anti–PD-1 treatment. By orthotopically injecting a developed Luc-SIY⁺ cell line, the study achieved a high metastatic burden (83%) in the spleen, lymph nodes, and pancreas by day 33 post-injection [33]. However, in the clinical setting, lungs and bones are the most common sites of bladder cancer metastasis [8, 9]. To overcome these limitations, we used GFP and Luciferase double-expressing reporter to label a syngeneic mouse bladder cancer cell line CMV-TRP (ex vivo transduction in urothelial cells from *Trp53*^*fl/fl*^ *Pten* ^*fl/fl*^ *Rb1* ^*fl/fl*^ *mice*) and delivered it into lateral tail vein to establish 100% mBC in lung or bones of C57 BL/6J mice within one month (Figs. 1 and 2). Using this real-time metastatic bladder cancer model, we demonstrated primary resistance to anti–PD-1 therapy (Fig. 3). Therefore, our model offers distinct advantages by more accurately recapitulating the clinical metastatic pattern.

PD-1/PD-L1 immune checkpoint inhibitors with chemotherapy or EV have shown clinical efficacy in 57–67% of patients with mBC [10, 11]. Intrinsic resistance in a significant proportion of patients, along with the emergence of acquired resistance in others, continues to limit both the durability and overall effectiveness of these therapies [34–36]. However, the mechanisms underlying resistance to immunotherapy in mBC remain poorly understood. Our mBC model showed no significant changes in tumor growth or the immune microenvironment following treatment with a mouse anti–PD-1 antibody, indicating that these metastatic tumors are immunologically “cold”. Although scRNA-seq data for lung metastases were not available, we re-analyzed the subcutaneous tumor datasets from the same anti–PD-1 treatment and same TKO tumor cells to further characterize the immune microenvironment and infer potential cellular features associated with immunotherapy resistance in metastatic lesions. The analysis in Supplementary Fig. S1 confirmed low percentage of most immune cells infiltration in the TKO tumor observed in IHC staining and suggested low expression of available immune checkpoint target genes panel may underlie the mechanisms driving anti–PD-1 resistance in TKO tumors. Low or absent PD-L1 expression in TKO tumors may also contribute to anti–PD-1 resistance, as a study in advanced gastroesophageal cancer reported that PD-L1 expression was the strongest predictor of overall survival benefit from immune checkpoint inhibitor (ICI) therapy [37]. Further studies are warranted to compare primary tumors with metastatic lesions to better understand site-specific differences contributing to immunotherapy resistance. Additionally, analyzing untreated versus treated samples will help elucidate the mechanisms underlying primary resistance to immune checkpoint blockade. Hence, we present a robust mouse model with primary immunotherapy resistance, providing a platform to study resistance mechanisms and test novel immunotherapies.

This model presents several limitations that should be considered. First, the acute distress observed following tail vein injection of 0.8 or 1 million cells reflects a technical limitation associated with high cell volume load. Although approximately 90% of mice recovered within 15 min, the transient morbidity observed during injection could be mitigated by reducing the cell dose (e.g., to 0.5 million) or by enriching for viable single cells. These adjustments would still support metastatic seeding while minimizing adverse effects. Alternatively, caudal artery injections may offer a more tolerable route for generating metastases [20], although it may not produce the same pattern of dissemination, including lung involvement that our model uniquely recapitulates. Second, the variability in metastatic tumor growth kinetics poses a challenge for synchronized therapeutic intervention. While the take rate is 100% by ~ 21 days post-injection, the onset and progression of detectable metastases vary between individual mice. A threshold of bioluminescence of 10^6^ photons/second prior to treatment initiation is a reasonable method that ensures tumor burden is sufficient for therapeutic assessment. Third, firefly luciferase reporters may introduce immunogenicity in syngeneic C57BL/6J mice, while GFP is only mildly immunogenic [38–40]. Future studies using unlabeled TKO cells will help exclude possible reporter-related immune effects. Fourth, this rapid metastatic model bypasses the initial steps of metastasis, such as local invasion and intravasation, and instead enters the circulation directly via the tail vein, which can’t mimic the full metastatic cascade. The fifth limitation is the modest sample size, which introduces minor statistical constraints; however, it did not influence the overall survival outcomes observed in our findings. Together, these limitations do not detract from the model’s overall utility for studying metastatic bladder cancer biology and immunotherapy resistance.

## Conclusion

In summary, our mBC model is not only highly clinically relevant but also serves as a robust platform for real-time tracking of metastatic dissemination and dissecting primary resistance to immune checkpoint blockade. It provides a powerful system for investigating the underlying mechanisms of metastatic progression and identifying actionable strategies to convert immunologically “cold” tumors into immune-targetable disease.

## Supplementary Information

Supplementary Table S1.

Supplementary Fig.S1

## Data Availability

No datasets were generated or analysed during the current study.

## Abbreviations

ANOVA: Analysis of Variance
ATP: Adenosine Triphosphate
BF: Bright Field (Microscopy)
BSC: Back Scatter
CAR-T: Chimeric Antigen Receptor T Cell
DMEM: Dulbecco’s Modified Eagle Medium
EDTA: Ethylenediaminetetraacetic Acid
EV: Enfortumab Vedotin
FACS: Fluorescence-Activated Cell Sorting
FSC: Forward Scatter
GC: Gemcitabine and Cisplatin
GEMM: Genetically Engineered Mouse Model
GFP: Green Fluorescent Protein
H&E: Hematoxylin and Eosin
IHC: Immunohistochemistry
IF: Immunofluorescence
IVIS: In Vivo Imaging System
mBC: Metastatic bladder cancer
MRI: Magnetic Resonance Imaging
MVAC: Methotrexate, Vinblastine, Adriamycin, and Cisplatin
PBS: Phosphate-Buffered Saline
PD-1: Programmed Cell Death Protein 1
PD-L1: Programmed Death-Ligand 1
PDX: Patient-Derived Xenograft
TKO: Triple Knockout
t-SNE: t-distributed Stochastic Neighbor Embedding
SNN: Shared Nearest Neighbor
