# Supplementary material for: Modeling primary immunotherapy resistance in metastatic bladder cancer: a syngeneic, bioluminescent mouse model
Source: Cancer Cell Int. 2026 Jan 8;26:73. doi: 10.1186/s12935-025-04117-x (PMC12879323; doi:10.1186/s12935-025-04117-x)
Supplement: Supplementary file 2 — Supplementary Fig.S1 [file 12935_2025_4117_MOESM2_ESM.pdf]

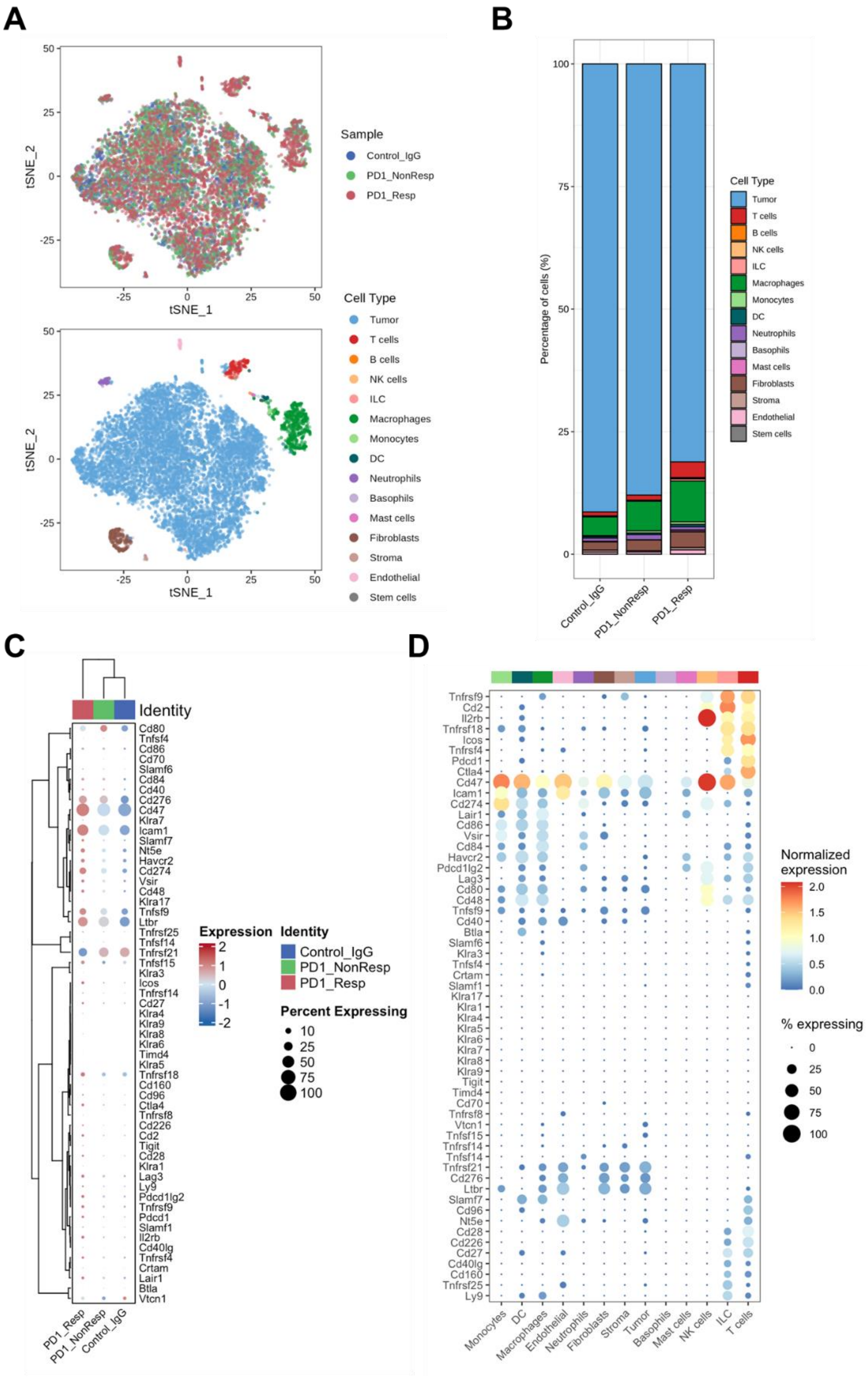

Supplementary Fig. S1 Immune microenvironment in TKO tumor treated with anti–PD-1 in subcutaneous model. (**A**) Transcriptomes of individual cells collected from TKO tumors projected in t-SNE space. Each cell is colored by sample (response to immunotherapy) or broad cell type annotation. (**B**) Bar plots showing the proportion of cell types (percentage of all cells) within each sample. (**C**) Scaled normalized gene expression of immunotherapy target genes listed by Shi *et al*. Data is presented as a dot plot for each gene across sample group, color coded for scaled expression level and size coded for percent of expressing cells. (**D**) Unscaled normalized gene expression of immunotherapy target genes within control IgG2a tumor. Data is presented as a dot plot for each gene across annotated cell type.
